# The role of Lymphadenectomy in early-stage mucinous Ovarian Cancer

**DOI:** 10.12669/pjms.41.10.12370

**Published:** 2025-10

**Authors:** Volkan Karatasli, Duygu Ayaz, Alaattin Karabulut, Muzaffer Sanci

**Affiliations:** 1 Volkan Karatasli Department of Gynecologic Oncology, University of Health Sciences Balikesir Atatürk City Hospital, Balikesir, Turkey; 2 Duygu Ayaz Department of Pathology, University of Health Sciences Tepecik Education and Research Hospital, Izmir, Turkey; 3 Alaattin Karabulut Department of Obstetrics and Gynecology, University of Health Sciences Tepecik Education and Research Hospital, Izmir, Turkey; 4 Muzaffer Sanci Department of Gynecologic Oncology, University of Health Sciences Tepecik Education and Research Hospital, Izmir, Turkey

**Keywords:** Early stage, Lymphadenectomy, Mucinous ovarian cancer

## Abstract

**Objectives:**

To assess the effect of lymphadenectomy on morbidity and survival in patients with early-stage mucinous ovarian cancer.

**Methodology:**

Patients with clinical stage-I primary mucinous ovarian cancer who underwent surgery in the gynecological oncology clinic of Tepecik Education and Research Hospital, Izmir, Turkey between January 1998 to December 2018 were analyzed retrospectively. The patients were divided into two groups according to whether lymphadenectomy (pelvic ± para-aortic) was performed or not. The groups were compared in terms of clinical variables.

**Results:**

Forty-five patients were examined. Lymphadenectomy was performed in 30 (66.7%) patients. Metastasis was not detected in any of the lymph nodes. There was no significant difference between the groups in terms of age, menopausal status, stage, tumor grade, bilaterality, ascites, tumor size and preoperative cancer antigen 125, carbohydrate antigen 19-9, carcinoembryonic antigen. Patients who underwent lymphadenectomy had longer durations of operation time and hospital stay (p<0.001 and p=0.011, respectively). The median follow-up time was 96 (3-240) months. Recurrence developed in two (4.4%) patients. The five-year disease-free survival rates of the groups with and without lymphadenectomy were 96.4% and 92.9%, respectively (p=0.666). The five-year overall survival rates of the groups with and without lymphadenectomy were calculated as 96.4% and 92.9%, respectively (p=0.666).

**Conclusions:**

The risk of lymph node metastasis is very low in early-stage mucinous ovarian cancer. Lymphadenectomy causes an increase in the duration of operation and hospital stay and does not contribute to disease-free and overall survival. Therefore, routine lymphadenectomy should be carefully evaluated in clinical early-stage mucinous ovarian cancer to reduce morbidity.

## INTRODUCTION

Mucinous ovarian cancer is a rare histological subtype among epithelial ovarian cancers.^1^ It usually presents with unilateral pelvic masses that have reached large dimensions in premenopausal patients and is detected at an early stage at the time of diagnosis.^2^ The prognosis is generally good in patients with early-stage disease.^1^

Complete surgical staging is recommended for the treatment of mucinous ovarian cancer.^3^ Part of staging is sampling or dissection of the lymph nodes.^3^ Although lymphadenectomy is recommended in clinical early-stage patients, the risk of lymph node metastasis is low, and the effect of systematic lymphadenectomy on survival is controversial.^2^,^4^,^5^ Although lymphadenectomy has been reported to have no effect on disease-free survival (DFS) and overall survival (OS) in stage IA/B disease, it has been emphasized that it has a positive contribution to survival in stage IC.^4^

In addition, it has been stated that lymphadenectomy increases the morbidity of patients, causing longer operation time and hospital stay in the short term, lymphedema and lymphocele in the long term.^6^,^7^ This study aims to evaluate the effect of lymphadenectomy on morbidity and survival of patients with early-stage mucinous ovarian cancer.

## METHODOLOGY

In this retrospective study, patients who underwent surgery in the gynecological oncology clinic of Tepecik Education and Research Hospital, Izmir, Turkey between January 1998 to December 2018 were examined. Patients who underwent surgery for primary mucinous ovarian cancer and had clinical stage-I disease findings were included in the study. Cases in advanced stage (stage ≥ II), with concomitant gynecological malignancy, metastatic tumor, and unavailable follow-up data were excluded from the study. Demographic characteristics (age, menopausal status) and clinical data of the cases (stage, grade, bilaterality, presence of ascites, tumor size, preoperative serum tumor markers, lymphadenectomy status, number of lymph nodes, operation time, hospital stay, presence of adjuvant treatment, recurrence status, DFS, and OS) were examined.

Patients with clinically suspicious adnexal masses were evaluated with abdominal computed tomography or magnetic resonance imaging. Operations were performed by a team of experienced gynecologic surgical oncologists. A midline laparotomy was performed with a vertical incision. Suspicious masses were sent for frozen section examination. Evaluations were performed by experienced gynecopathologists. Comprehensive staging with omentectomy, peritoneal biopsies and washings was performed. Gastrointestinal evaluation was applied by colonoscopy, upper endoscopy and intraoperative examination to rule out primary gastrointestinal tumors.

The staging was performed in accordance with the 2014 International Federation of Gynecology and Obstetrics staging system.^8^ Postoperative findings were discussed in a multidisciplinary tumor board and adjuvant treatment was decided according to the guidelines.^9^ Adjuvant chemotherapy (paclitaxel-carboplatin, or 5-fluoruracil with leucouverin and oxaliplatin, or capecitabine and oxaliplatin) was decided based on existence of surgical spill, preoperative capsule rupture, tumor on the ovarian surface, positive peritoneal cytology, bilaterality, grade and growth pattern of tumors and performance status of the patient. The treatment was applied with patient acceptance. Patients were divided into two groups according to whether lymphadenectomy (pelvic ± para-aortic) was performed or not. The groups were compared in terms of clinical variables.

In the follow-up of the patients, check-ups were performed every three months for the first two years, every six months for the next three years, and annually thereafter. Vulvovaginal and pelvic examinations were performed at each outpatient visit, and imaging methods were performed when recurrence was suspected. Newly emerged and confirmed lesion was defined as recurrence. The time of recurrence after primary surgery, or in the absence of recurrence until the last follow-up was defined as DFS. OS was defined as the time from surgery to death or final follow-up.

### Ethical Approval:

The institutional local ethics committee approval (Number: 2020/4-9; Dated: March 11, 2020) was obtained for the study.

### Statistical analyses:

It was performed with Statistical Package for the Social Sciences (SPSS) version 22 (Chicago, IBM Corp.). Demographic data were presented with descriptive statistics. Clinical variables were compared with the Mann Whitney-U test and chi-square tests. The Kaplan-Meier method was used for survival analysis. P<0.05 was considered statistically significant.

## RESULTS

Forty-five patients were included in the study. Thirty (66.7%) patients underwent lymphadenectomy (pelvic in eight patients, pelvic and para-aortic in 22 patients). The median number of dissected pelvic lymph nodes was 16 (7-43), while that of the para-aortic lymph nodes was 6 (5-32). No metastases were detected in any of the lymph nodes. When the groups that underwent lymphadenectomy and did not undergo lymphadenectomy were compared, no significant difference was found between the groups in terms of age, menopausal status, stage, tumor grade, bilaterality, ascites, tumor size, and preoperative cancer antigen 125 (CA 125), carbohydrate antigen 19-9 (CA 19-9), carcinoembryonic antigen (CEA) (Table-I).

**Table-I:** Demographic and clinical features of the cases.

	Lymphadenectomy group n = 30, (%)	Non-lymphadenectomy group n = 15, (%)	p value
Age (years), mean ± SD	51.0 ± 15.1	54.9 ± 17.5	0.439
≤ 50	18 (60)	6 (40)	
> 50	12 (40)	9 (60)	0.205
***Menopausal status***			
Premenopausal	14 (46.7)	4 (26.7)	0.197
Postmenopausal	16 (53.3)	11 (73.3)	
***Stage***			
1A	21 (70.0)	11 (73.4)	0.314
1B	1 (3.3)	2 (13.3)	
1C	8 (26.7)	2 (13.3)	
***Grade***			
I	20 (66.7)	10 (66.7)	1.00
II/III	10 (33.3)	5 (33.3)	
Bilaterality	4 (13.3)	2 (13.3)	1.00
Ascites	4 (13.3)	1 (6.7)	0.507
Tumor diameter (cm), median (range)	22 (7-45)	18 (8-35)	0.468
CA 125 median (range)	46.5 (3-799)	30 (9-220)	0.181
CA 19-9 median (range)	58.5 (1-1579)	29.0 (3-700)	0.339
CEA median (range)	7 (0.7-25)	5 (0.1-126)	0.780
Operation time (min), mean ± SD	173.0 ± 38.0	105.3 ±32.0	**<0.001**
Duration of hospital stay (days), median (range)	5.5 (3-10)	5 (3-7)	**0.011**
Adjuvant chemotherapy	16 (53.3)	9 (60.0)	0.671
Recurrence	1 (3.3)	1 (6.7)	0.609

SD = standard deviation; CA 125 = cancer antigen 125, U/mL; CA 19-9 = carbohydrate antigen 19-9, U/mL, available in 22 cases; CEA = carcinoembryonic antigen, U/mL, available in 20 cases; min = minutes; p<0.05 = bold.

The duration of operation and hospital stay were longer in patients who underwent lymphadenectomy (p < 0.001, p = 0.011, respectively). While there was no microscopic disease in omentum and peritoneal biopsies, positive peritoneal cytology was detected in three cases. Three patients underwent fertility-sparing surgery (unilateral salpingo-oophorectomy). While three patients were in the infiltrative pattern, 24 patients were in the expansile type category (available in 27 cases). Adjuvant chemotherapy was applied in 53.3% of the lymphadenectomy group and 60% of the non-lymphadenectomy group. The median follow-up time was 96 (3-240) months. Recurrence developed in two (4.4%) patients (Table-II). The first case presented with diffuse peritoneal carcinomatosis at the 24th month after adjuvant chemotherapy and died at 35th month despite systemic chemotherapy. The second case with an infiltrative tumor presented with liver parenchymal metastasis in the eighth month of follow-up without adjuvant treatment after surgery and died after the first dose of systemic chemotherapy.

**Table-II:** Characteristics of cases with recurrence.

	Age (years)	CA 125	Type of operation	Tumor diameter (cm)	Stage	Adjuvant therapy	Time (months) and site of recurrence	Therapy for recurrence	Status and overall survival (months)
Case 1	75	120	TAH+BSO	22	IA	6 cycles of CT	24, abdomen	CT	Died of disease, 35
Case 2	77	11	TAH+BSO+ PLND	20	IA	(-)	8, liver	CT	Died of disease, 8

CA 125 = cancer antigen 125, U/mL; TAH+BSO = total abdominal hysterectomy, bilateral salpingo-oophorectomy, PLND = pelvic lymphadenectomy; CT = chemotherapy.

**Fig.1:**
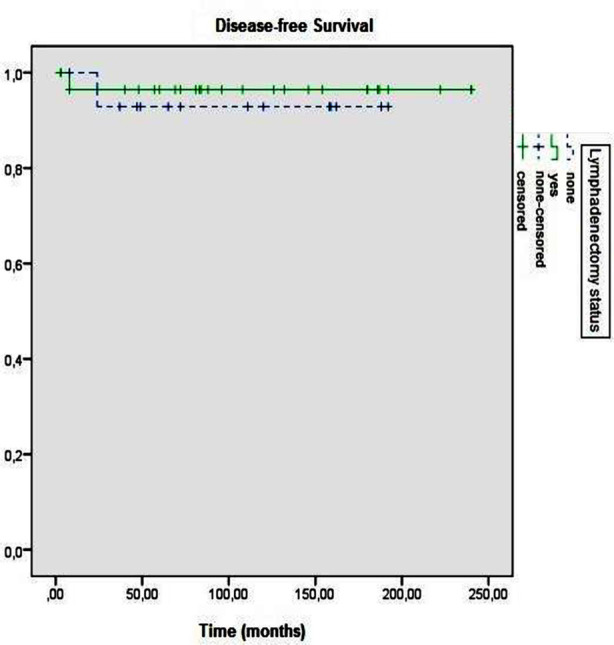
Disease-free survival according to lymphadenectomy status (p = 0.666).

**Fig.2:**
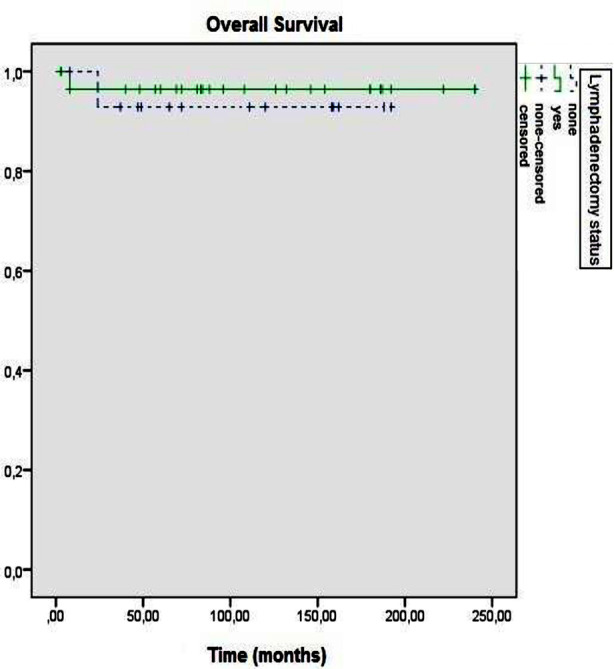
Overall survival according to lymphadenectomy status (p = 0.666).

Both five-year DFS and OS rates of the cases were 95.2%. The five years DFS rates of the groups with and without lymphadenectomy were 96.4% and 92.9%, respectively (p=0.666) (Fig.1). The five years OS rates of groups with and without lymphadenectomy were calculated as 96.4% and 92.9%, respectively (p=0.666) (Fig.2).

## DISCUSSION

In this study, we evaluated the effect of lymphadenectomy on morbidity and survival in patients with clinical early-stage mucinous ovarian cancer treated at a tertiary cancer center over a 20-years period. Our findings showed that no lymph node metastasis was detected in any patient who underwent lymphadenectomy. Moreover, lymphadenectomy did not improve DFS or OS, but was associated with increased morbidity, including longer operation time and hospital stay. These results are consistent with previous reports from international cohorts and systematic reviews, which also demonstrated very low rates of nodal metastasis and no significant survival benefit from systematic lymph node dissection in early-stage mucinous ovarian cancer.^10^-^15^ At the same time, our study contributes additional evidence by providing long-term follow-up data from a single center, further supporting the consideration of omitting routine lymphadenectomy in selected patients to reduce morbidity.

Mucinous ovarian cancer is a subtype of epithelial ovarian cancer that is usually diagnosed at an early stage and has a good prognosis.^2^,^10^ Although lymphadenectomy is recommended as part of surgical staging, the risk of lymph node metastasis in clinical early-stage disease is very low.^6^,^11^ In a systematic review, 278 patients with early-stage mucinous ovarian cancer were examined, and it was stated that lymph node dissection did not have an impact on staging.^12^ In addition, it was determined that lymphadenectomy did not contribute to DFS and OS.^13^-^15^

Compared to other histological types, mucinous ovarian cancer metastasizes to lymph nodes less frequently, especially in the early stages.^13^,^16^ In a study examining 1,602 patients with clinical early-stage mucinous ovarian cancer, the rate of lymph node metastasis was found to be 1.7%.^4^ In a systematic review of seven studies, the rate of lymph node metastasis was reported as 0.7%.^12^ In our study, no metastasis was found in the dissected lymph nodes, as found in Salgado et al.^6^ Age is an independent risk factor for patients with mucinous ovarian carcinoma.^17^ More complex surgery can be performed in young patients with early-stage disease findings.^4^,^18^

In their study, Nasioudis et al. found that patients who underwent lymphadenectomy were at an earlier age than those who did not.^4^ However, consistent with the findings in our study, Yoshihara et al. did not detect any age difference between the two groups.^13^ It has been reported that the tumor size is larger in patients who underwent lymphadenectomy.^4^, ^19^ Salgado et al. reported that tumor size was not different between the two groups, which is consistent with our study.^6^ Tumor markers such as CA 125, CEA and CA 19-9 may be useful in the diagnosis and follow-up of mucinous ovarian tumors.^20^ Preoperative CA 19-9 and CEA were not different between those who underwent lymphadenectomy and those who did not, which is also in accordance with our findings.^6^ It was also stated that the level of CA 125 was lower in those who underwent lymphadenectomy.^6^ In our study, there was no difference between CA 125 levels, which is consistent with another study.^13^

It was emphasized that lymph node dissection should be avoided to avoid surgical complications of lymphadenectomy such as longer operation time and blood loss.^6^,^7^ In the present study, the duration of operation and hospital stay were longer in patients who underwent lymphadenectomy. Also in the longer term, lymphoceles and lymphedema may lower the quality of life in patients undergoing lymphadenectomy.^21^

Recurrence occurs less frequently in mucinous ovarian cancer at an early stage compared to other histological types.^2^ Yoshihara et al. reported the recurrence rate as 11.8%,^13^ while Salgado et al. reported it as 5.5%.^6^ In our study, the recurrence rate was found to be lower than those reported by previous studies. Nasioudis et al. reported that lymphadenectomy improved five years survival in stage-I patients.^4^ In their study, in which no metastasis was found in any patient who underwent lymphadenectomy, Salgado et al. stated that lymphadenectomy did not change recurrence, DFS, and OS, and that routine lymphadenectomy may not be performed.^6^ In another study, it was emphasized that lymphadenectomy was not a prognostic factor.^13^ Lymph node dissection did not contribute to DFS and OS in the present study.

Recently, Jin and Zhou published an updated systematic review and meta-analysis, reporting that lymphadenectomy in early-stage epithelial ovarian cancer may improve overall and progression-free survival, although the retrospective nature and heterogeneity of included studies necessitate cautious interpretation.^22^ Our findings, however, suggest that in the mucinous subtype, the risk of lymph node metastasis is exceedingly low, and no survival benefit was observed despite increased morbidity associated with the procedure.

The strength of our study lies in the inclusion of patients treated at a single tertiary cancer center with more than two decades of gynecologic oncology experience, comprehensive surgical staging, and long-term follow-up (median 96 months). All histopathological evaluations were performed by experienced gynecopathologists, ensuring diagnostic reliability. Nonetheless, the retrospective design and relatively small sample size, inherent to the rarity of early-stage mucinous ovarian cancer, remain important limitations. Despite these constraints, our study provides novel and clinically relevant evidence indicating that systematic lymphadenectomy may not be necessary in clinical stage I mucinous ovarian cancer, as it increases perioperative morbidity without improving survival outcomes. Future multicenter prospective studies and randomized controlled trials with larger cohorts are warranted to validate these findings and guide evidence-based surgical management in this rare subtype.

### Limitations:

The main limitations of this study are its retrospective design, single-center setting, and relatively small sample size. However, the strengths include long-term follow-up and pathology review by experienced gynecopathologists, which enhance the reliability of the results.

## CONCLUSION

The risk of lymph node metastasis in early-stage mucinous ovarian cancer is very low. Lymphadenectomy causes an increase in the duration of operation and hospital stay and does not contribute to DFS and OS. Therefore, routine lymphadenectomy should be carefully evaluated in clinical early-stage mucinous ovarian cancer to reduce morbidity.

## Data availability:

The data that support the findings of this study are available from the corresponding author upon reasonable request.

### Author’s Contribution:

**VK:** Conceived, designed and did statistical analysis & manuscript writing, is responsible for integrity of research.

**DA, AK:** Did data collection, literature search, data analysis and editing of manuscript.

**MS:** Critical review, analysis**,** and final approval of manuscript.
